# A Case of Chronic Pneumonia, with Anomalous Physical Signs—Abscess

**Published:** 1850-04

**Authors:** David W. Yandell

**Affiliations:** Louisville, Ky., Attending Physician for the Faculty at the Louisville Marine Hospital


ART. III.—*A Case of Chronic Pneumonia, with Anomalous Physical Signs.—Abscess.—Death.—Autopsy.* By DAVID W. YANDELL, M. D., of Louisville, Ky., Attending Physician for the Faculty at the Louisville Marine Hospital.

Patrick Green, marine, æt. 30, an Irishman and a hard drinker, was admitted into the hospital on the 4th of September, 1849. His general health, according to his own account, had been singularly good for one of his occupation and habits. In May last, while lifting a bale of cotton on the levee in New Orleans, he was suddenly seized with a severe pain in the right side of the chest, which was soon followed by a high fever and frequent dry cough. He entered one of the New Orleans hospitals; was cupped and blistered over the seat of the pain; took medicines internally, and at the end of a week was so far relieved as to be able to resume his work. The cough, however, still continued. It appeared to him that after this attack he was particularly liable to contract cold on the slightest exposure, when the pain and fever would return and the cough increase. His general health became impaired; his appetite was poor and capricious; he lost flesh and strength and spirits. His cough continued to grow more and more troublesome; was especially severe early in the morning, and often kept him from closing his eyes during the night.

The cough was dry, from May until the first of August, when it was followed by the expectoration of a thick, straw-colored matter. Very shortly after this, hectic fever and night sweats came on.

On the 1st of September he was exposed to cold; had a chill. This was followed by fever, pain in the right breast, and aggravation of the cough. The next morning, on getting up, the patient remarked an entire change in the character of the sputa. They were red, bloody, mucous, not very tenacious, and very abundant. Two days after this, he was received into the hospital, when the following condition of things was observed:

Expression languid and weary. Skin dry; pulse eighty eight, irritable; tongue slightly coated; bowels regular; no appetite; thirst; cough frequent, paroxysmal and painful; respiration twenty-three, short; expectoration copious, raised at each paroxysm of cough, of a very disagreeable earthy odor. The color was striking and peculiar. It was pink. The blood was intimately admixed with the other matters, save that here and there, there was a streak of muco-purulent fluid.

*Physical signs.*—A shade of dullness was observed on the right side, from the third rib downwards. Left side resonance and respiratory murmur normal. Respiratory murmur at the superior part of the right side normal.—After passing the third rib it becomes mingled with the mucous ronchus and occasional friction sounds. The ronchus is most distinct posteriorly, while in this region the friction sound is entirely wanting. There is also a slight, deep-seated and indistinct crepitation at the posterior part of the right side.

I ordered four cups on the anterior and four on the posterior part of the chest over the seat of the pain, and the use of the following cough mixture:

℞. Syrup tolutan., ℥vj.
Tinct. digital., ʒj.
Vin. ipecac. ℥iv.
Tinct. opii, ℥ss.
Sol. acac. ℥iv.

M. S. Dose, ʒj. every two or three hours.

On the next day, the patient expressed himself free from pain. The physical signs remained the same.

During the next eight days there was no perceptible alteration in the condition of the patient. The cough had, in some degree, perhaps, abated. The expectoration had in no wise changed its character nor diminished in quantity. The patient was daily spitting up from one and a half to two spittoons, of the ordinary size, full, during the twenty-four hours.

Deeming the use of astringents advisable, the patient was directed to take the acetate of lead, in combination with opium, though without any apparent relief being obtained.

The hectic fever and night sweats were greatly mitigated in intensity by the administration of the remedies usual in such cases. Day by day, however, the patient grew more and more feeble; his appetite was totally lost; he was scarcely master of enough strength to walk about the wards. His cough distressed him by day, and kept him awake at night. He had occasional diarrhea, and once or twice every week exhausting night sweats. Still the physical signs remained fixedly the same. Blisters were used; permanent counter-irritation; cups when pain was complained of; tonics, restoratives, nutricious food, and finally cod liver oil. This was in September and October. Towards the close of the latter month, while calling the attention of the young gentlemen present at the hospital clinique to the case, I heard unequivocal signs of the existence of a cavern in the mammary region of the right side. This was while the patient was lying down. When, at my request, he sat up, all cavern signs had entirely disappeared, and were never after perceptible, notwithstanding careful search was made almost every morning, from that time until the patient's death.

After one month of unavailing treatment by cups, blisters, opium, ipecac, and emetic tartar, it was deemed advisable to bring the system of the patient gently under the influence of mercury. This was effected by the administration of two grains of blue mass, and quarter of a grain of opium, three times a day, and the mercurial ointment dressing to the blisters, and was kept up for a fortnight. This proved quite as inefficient as the former remedies had done. The cod liver oil was now exhibited in combination with the syrup of iodide of iron, and the blisters repeated. The oil produced such irritation of the bowels that it was suspended after using it ten days. The iodide of iron was continued however for three weeks. As high as twenty-five drops of the syrup of iodide of iron was given three times a day, without producing any sensible change in the condition of the patient.

During the second, third, and fourth week in November, the sugar of lead in doses of one grain in combination with half grain of opium was given every six hours. The expectoration seemed for a few days to be lessened in quantity—its characters were uniformly the same. Other astringents were taken without appreciable effect. Two months had passed away, and the third was half advanced when the only noticeable change in the physical signs was an increase in the dullness of the right side inferior to the third rib, and a greater feebleness of the respiratory murmur over same region. And perhaps the broncophony was more distinct than it had been before—though it was very clear and loud from the first.

Emetics of ipecac and sulphate of copper were now exhibited every other morning. Doing no apparent good, and being bitterly complained of by the patient, they were omitted, after the fourth had been taken.

The patient at this date, 15th of December, lies entirely on the left side. Lying on either his back or right side increases cough. Within a few days past has had considerable dyspnea on the right side. Expectoration copious and fetid. Allowed brandy and water several times a day.

Gradually the patient continued to wear away. His strength steadily failed. His appetite was entirely lost. The pain in the side recurred every day or two. The dyspnea slowly augmented. The cough harassed him constantly. Hectic, night sweats, the copious expectoration and an abundant diarrhea, which more than twenty remedies had failed to arrest, eventually exhausted the remaining powers of the patient, and on the 5th of January, just four months after I had first seen him, he died.

*Autopsy twelve hours after death.*—Left lung healthy. The whole of the right lung, save a small portion of the superior lobe, not larger than a man's fist, which was healthy, was hepatized. Both varieties of hepatization were present, the red at the superior and middle portions of the lung, the grey at the lower. An abscess, about the size of a hen egg was found near the surface of the lung just opposite the point where the cavern signs had been detected during life. It was filled with a very thick tenacious pus. Another abscess, of five times the volume of this, was formed on a direct line with it at the base of the lung, and was formed by the lung, diaphragm and liver, one third of it occupying the superior surface of the liver, one third lay between the liver and lungs, its walls being formed by the diaphragm and false membrane, while the remaining third was situated in the pulmonary substance. The contents of this were identical in every particular with those of the first. Towards the summit of the right lung where the friction sounds had been heard during life, numerous patches of lymph and traces of a recent pleurisy were found. Remaining organs of body healthy.

Louisville, Ky., March 20, 1850.

